# Complete response to orally administered melphalan in malignant pleural effusion from an occult female genital organ primary neoplasm with *BRCA1*/*2* mutations: a case report

**DOI:** 10.1186/s13256-018-1674-3

**Published:** 2018-05-06

**Authors:** Frank S. Fan, Chung-Fan Yang

**Affiliations:** 1 Section of Hematology and Oncology, Department of Medicine, Ministry of Health and Welfare Changhua Hospital, Chang-Hua County, Taiwan, Republic of China; 2 Department of Pathology, Ministry of Health and Welfare Changhua Hospital, Chang-Hua County, Taiwan, Republic of China; 3 Department of Administration, Ministry of Health and Welfare Changhua Hospital, 80, Sec. 2, Chung-Jeng Rd, Pu-Shin Township, Chang-Hua County, Taiwan, Republic of China

**Keywords:** Malignant pleural effusion, Ovarian cancer, *BRCA1*/*2* mutation, Melphalan chemotherapy

## Abstract

**Background:**

Definite diagnosis of metastasis from unknown primary depends on a comprehensive immunohistochemical investigation of tumor specimen. Accurate identification of the origin site usually helps a lot in choosing the most appropriate treatment. Molecular characterization provides more chance of a cure. Echoing modern medical development, *BRCA1*/*2* mutations have been correlated with high efficiency of poly(adenosine diphosphate-ribose) polymerase inhibitors in ovarian cancer. While a previous case report demonstrated a surprising cure of platinum-resistant ovarian cancer with *BRCA2* mutation by orally administered melphalan.

**Case presentation:**

A 53-year-old Taiwanese woman’s malignant pleural effusion was diagnosed to be a metastasis from an occult cancer in female genital organ by diligent pathological study despite absence of image evidence. She resolutely refused intravenously administered chemotherapy. After failure of anti-estrogen tamoxifen, orally administered melphalan achieved excellent complete remission. Pathogenic homozygous *BRCA1* and *BRCA2* mutations were later detected in tumor cells by next-generation sequencing. The same *BRCA2* mutation exists in a heterozygous status in the germline deoxyribonucleic acid.

**Conclusions:**

This is so far the second report of long-term remission of advanced female genital organ cancer with *BRCA* mutations achieved by orally administered melphalan. *BRCA1*/*2* mutations and even all “BRCAness” of malignancy, at least ovarian cancer and ovarian-related cancers, probably not only correlate with high efficacy of poly(adenosine diphosphate-ribose) polymerase inhibitors but also lead to a high-potential cure by orally administered melphalan. We recommend that clinical trials that test this assumption be carefully designed and sophisticatedly performed.

## Background

The diagnosis and management of metastatic carcinoma from unknown primary site remain a challenge to both pathologist and oncologist. Poor prognosis is especially recognized as a feature of adenocarcinoma of unknown origin with pleura metastasis [1]. A systemic approach with intensive image and cytopathology study including extended immunohistochemistry staining has established a reliable diagnostic algorithm for clinicians to make the most appropriate therapeutic decision for malignant pleural effusion from unknown origin [2]. We present here a middle-aged woman whose massive pleural effusion was determined to be a metastasis from an undetectable genital organ site based on cytology immunostains. Although the standard combination chemotherapy would be platinum and taxane for epithelial ovarian cancer and its related malignancies [3], she refused intravenous chemotherapy injection and took orally administered melphalan as the sole treatment after failure of anti-estrogen tamoxifen. To our surprise, her disease turned out to have a complete response with all the serum tumor markers returning to normal range. We later did a genetic investigation of her tumor sample by next-generation sequencing (NGS) technique and found pathogenic mutations of both *BRCA1* and *BRCA2*. The Fanconi anemia/BRCA pathway is a regulator of deoxyribonucleic acid (DNA) repair in response to double-strand breaks. Deficiency of either *BRCA1* or *BRCA2* leads to genomic instability and induces familial cancer [4]. We aim to discuss and bring attention to the remarkable therapeutic potential of melphalan for female genital organ cancer with *BRCA1*/*2* mutations.

## Case presentation

A 53-year-old married Taiwanese woman was brought to our emergency unit with the chief complaint of shortness of breath and chest tightness for 1 day in July 2016. Her breathing sound was diminished over the right lung field but there was no fever, jugular vein engorgement, superficial lymphadenopathy, heart murmur, or peripheral edema. She was not a cigarette smoker and denied alcohol abuse. Her type 2 diabetes mellitus and hyperlipidemia had been under regular medical control for 2 years. Her surgical history included removal of an intracranial aneurysm in 2003 and uvulopalatopharyngoplasty for relieving obstructive sleep apnea syndrome in 2005. Malignancy had never been diagnosed either in our patient or her family members in the past. There was no evidence of myocardial ischemia by electrocardiogram and cardiac enzyme examination.

A routine chest X-ray film revealed massive right-side pleural effusion pushing her mediastinum and heart shadow leftward. After admission to a ward, a pigtail catheter was inserted for effusion drainage. A laboratory study disclosed an exudate nature without signs of bacterial infection. A cytology investigation reported a metastatic adenocarcinoma. A DNA study did not discover gene mutations of epidermal growth factor receptor. Positive findings of serum tumor makers included cancer antigen 125 (CA 125) 89.6 IU/ml and cancer antigen 15–3 (CA 15–3) 43 IU/ml, respectively. A computed tomography (CT) scan of her chest and abdomen, however, did not detect any suspicious primary site of her malignancy. Since her respiratory distress had been relieved by therapeutic thoracentesis, she was discharged without any other treatment on her own terms.

**Fig. 1:**
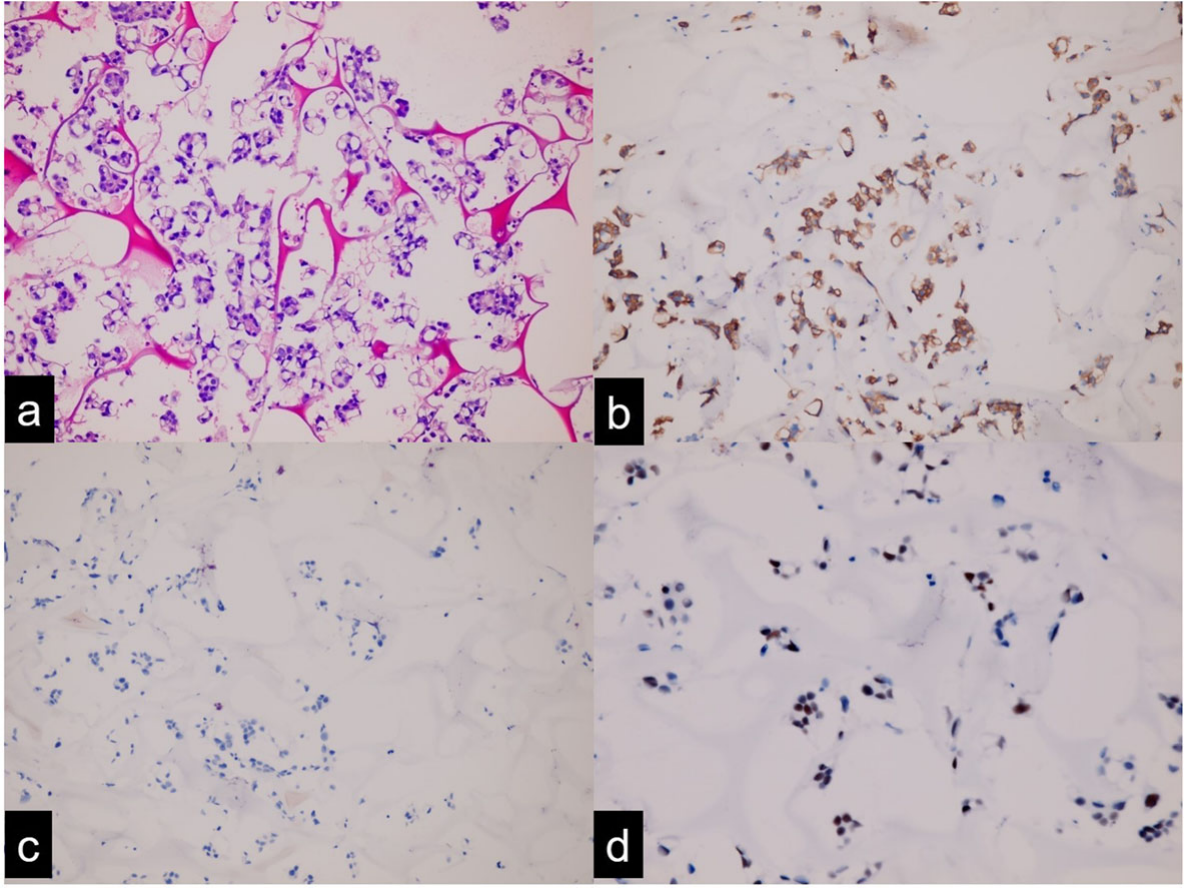
Cell block from pleural effusion shows metastatic adenocarcinoma, hematoxylin and eosin staining (**a**), with immunohistochemical results positive for cytokeratin 7 (**b**), negative for cytokeratin 20 (**c**), and scattered positive for paired-box gene 8 (**d**)

**Fig. 2:**
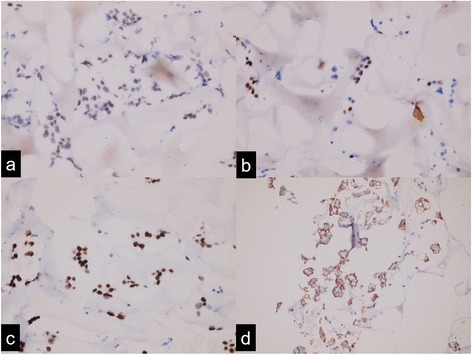
Immunohistochemical staining reveals carcinoma cells in pleural effusion to be mild positive for estrogen receptor (50%) (**a**), moderate positive for progesterone receptor (10%) (**b**), strongly positive for Wilms tumor 1 (100%) (**c**), and cancer antigen 125 (100%) (**d**)

A mammogram was arranged for her at an out-patient clinic in August 2016. The result was also negative while her pleural effusion began to recur as revealed on chest films. She was lost to follow up in the following 4 months and similar difficult breathing struck again eventually in December 2016. During her second hospitalization, her respiratory discomfort was rapidly eliminated by a second thoracentesis. The obtained pleural effusion was sent for cytologic examination and made into a paraffin-embedded cell block for subsequent immunohistochemical study. The cytomorphology under microscope was an adenocarcinoma that showed a gland-like structure or tight clusters composed of tumor cells with highly pleomorphic nuclei and markedly vacuolated cytoplasm (Fig. 1a). Extensive immunohistochemistry staining of the cell block prepared from the pleural effusion sediment in an attempt to find out tumor origin showed that the metastatic adenocarcinoma was positive for cytokeratin 7 (CK7) and negative for cytokeratin 20 (CK20). Other positive markers included paired-box gene 8 (PAX8; scattered), estrogen receptor (ER; mild, 50%), progesterone receptor (PR; moderate, 10%), Wilms tumor 1 (WT1; strong, 100%), p53 (moderate, 30%), and CA 125 (strong, 100%; Figs. 1 and 2). While human epidermal growth factor receptor 2 (HER-2), thyroid transcription factor 1 (TTF-1), napsin A, GATA binding protein 3 (GATA3), and gross cystic disease fluid protein 15 (GCDFP-15) turned out to be negative. These results led to a conclusion that our patient’s pleural metastasis came from an occult primary site in her female genital organs, such as ovary, fallopian tube, peritoneum, or endometrium according to published guidelines and diagnostic flow charts [5, 6]. Her serum CA 125 and CA 15–3 levels rechecked at the second admission were 58.1 and 81.9 IU/ml, respectively. A sonogram of her breast did not reveal any suspicious lesions.

**Fig. 3:**
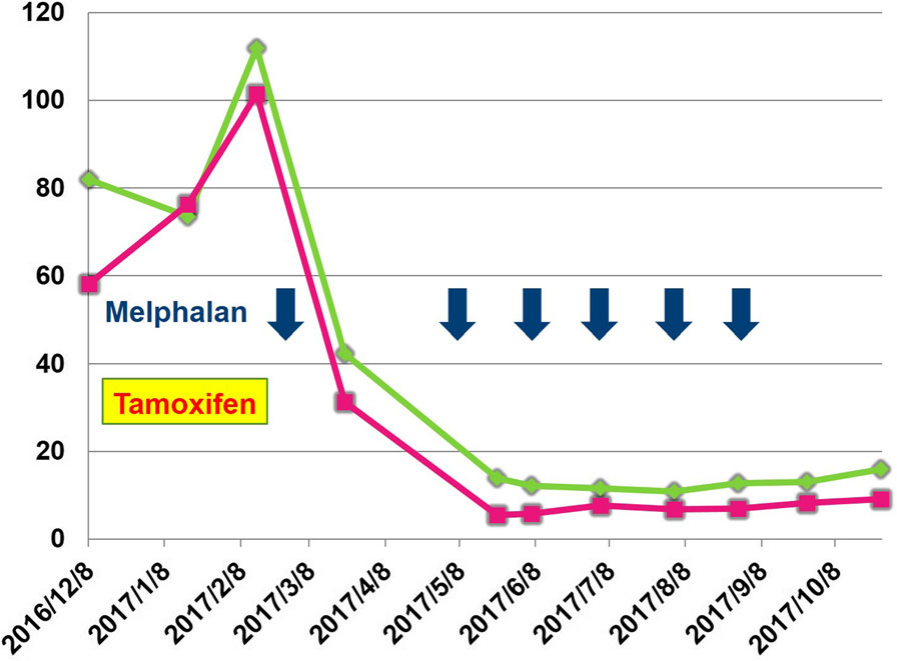
Change of serum tumor markers cancer antigen 15–3 (*green*) and cancer antigen 125 (*red*) along treatment course. *Yellow box*: The period of tamoxifen usage. *Deep blue arrow*: Each course of melphalan therapy

**Fig. 4:**
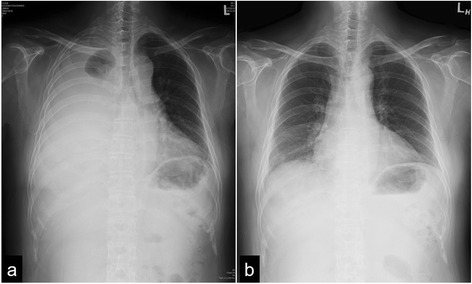
Chest X-ray films before indwelling drainage plus melphalan therapy (**a**, Feb. 14, 2017) and 2 months after completing six courses of melphalan treatment (**b**, Oct. 26, 2017)

Because she insisted that she did not want to receive any kind of intravenous chemotherapy, we let her take tamoxifen as the first step of treatment. Unfortunately, her disease was proven resistant to tamoxifen 2 months later and a third pigtail catheter drainage for massive pleural effusion had to be performed in February 2017. Orally administered melphalan (2 mg/tablet, Alkeran™; Excella GmbH; Feucht, Germany), 7 mg/m^2^ daily for 5 consecutive days, was substituted for rescue. The drainage catheter had to be removed due to an accidental rupture of the collecting bag 11 days after starting melphalan treatment and a carbuncle developed around the insertion site of the catheter a few days later. However, surprisingly, her serum tumor markers declined quickly, and the effusion disappeared in a short period of time. Although a CT scan from chest downward to pelvis once again failed to locate a definite primary site as before, she tolerated the therapy very well and remained disease free for 3 months after completing six courses of a 28-day cycle of melphalan treatment totally with the second course delayed for 4 weeks due to her own choice (Figs. 3 and 4). A CT scan performed in September 2017, 1 month after the sixth cycle of melphalan treatment, disclosed no evidence of recurrence.

Based on our curiosity and interest, we searched for *BRCA1* and *BRCA2* gene mutations in the DNA extracted from paraffin-embedded cell block of effusion by NGS analysis. The specimen was sent to Yourgene Genomics Core Laboratory (Yourgene Bioscience; Taiwan), which has Ion AmpliSeq™ Exome Certified Service, for targeted genome sequencing. Tumor genomic DNA was extracted and purified by RecoverAll™ Total Nucleic Acid Isolation Kit for formalin-fixed paraffin-embedded (FFPE; AM1975; Thermo Fisher Scientific). A targeted capture library was constructed according to the protocol QIAseq Targeted DNA panels for human *BRCA1* and *BRCA2* (DHS-102Z, QIAGEN). Template preparation and Ion 540™ Chip were performed on Ion Chef™ (4484177, Thermo Fisher Scientific) using respectively 120 pM of equimolar pooled libraries. The samples were sequenced on an Ion S5 XL System (A27214, Thermo Fisher Scientific) using 500 flows. Sequencing data were analyzed by Torrent Suite 5.2.1 (Thermo Fisher Scientific). Variant identification was performed using QIAGEN’s QIAseq targeted sequencing data analysis portal (http://ngsdataanalysis.sabiosciences.com/QIAseqDNA/). Accordingly, two significant homozygous complementary DNA (cDNA) mutations were detected: c.3214delC of *BRCA1* and c.5164_5165delAG of *BRCA2*. Our patient’s saliva was subsequently sent to a Clinical Laboratory Improvement Amendments (CLIA)-certified laboratory (CurieMed Clinical Laboratory, a Taiwan agent of Veritas Genetics, USA) available for sequencing of germline *BRCA1* and *BRCA2*. We collected and preserved 2 ml of saliva using SD-2000-DX collection kit (Samplify Bio, Massachusetts, United States of America). Genomic DNA extraction and purification, and targeted capture library DNA construction were performed by Veritas Genetics proprietary hybrid capture-based assay. The sample was sequenced on an Illumina NextSeq 500 platform. A heterozygous c.5164_5165delAG mutation of *BRCA2* was detected.

## Discussion and conclusions

Initially, in the absence of any image clues, our patient’s malignant pleural effusion should be diagnosed as a metastatic carcinoma of unknown primary. Nevertheless, intensive immunohistochemistry staining using a profile of valuable markers enabled differential diagnosis of the primary site to accurately focus on female genital organ. Although the tumor cells were positive for both ER and PR to a certain degree, negative results of GATA3 and GCDFP-15 might be able to exclude an occult primary in the breast due to their specificity for breast cancer [7]. On the other hand, strong positivity of PAX8 and WT1 are characteristic for epithelial carcinoma from female genital tract, especially the serous type [8, 9]. Thus we feel rather confident to define the primary site as an occult “pelvic serous tumor,” most likely a high-grade serous carcinoma of ovary, fallopian tube, or peritoneum. Combined detection of serum tumor markers CA 125 and CA 15–3 has previously been found to be quite sensitive for a diagnosis of ovarian cancer and is compatible with the case of this patient [10].

Traditional management of malignant pleural effusion emphasizes palliative modalities, such as therapeutic aspiration, indwelling catheter drainage, and pleurodesis [11]. However, with an effective systemic therapy, this distressing condition can be eliminated as part of the cancer itself. Although the average magnitude of ER expression is weaker in ovarian cancer cells than in breast cancer cells, the 8% objective response rate of tamoxifen in platinum-resistant ovarian cancer makes hormone therapy still a reasonable choice for this disease [12], particularly given the benefits of low toxicity, low cost, and convenient oral administration [13]. Unfortunately, this therapeutic policy did not succeed in the present case.

Over the past 3 decades, standard first-line chemotherapy for ovarian cancer has evolved through clinical trials to be a combination of platinum and taxane, such as a carboplatin/paclitaxel regimen [14]. The oldest single agent melphalan has retreated from the frontline and become a palliative modality for advanced platinum-resistant ovarian cancer with a best response rate of 30% in a small retrospective study [15]. By contrast, when used up front for advanced ovarian cancer, melphalan alone achieved a response rate of 47% (20% complete, 27% partial) and the 5-year survival rate for responders was 16% [16]. What we are curious about is this exciting long-term survival rate. Is there any underlying cause to make these 16% patients so lucky?

Fanconi anemia core complex coordinate with BRCA1 and BRCA2 to initiate homologous recombination and DNA interstrand crosslink repair. Deficiency of this Fanconi anemia/BRCA pathway promotes tumorigenesis but also makes cancer cells sensitive to chemotherapy agents which act by crosslinking DNA, while alkylator melphalan is just one of such agents [17]. Research work in the laboratory has demonstrated that a robust Fanconi anemia/BRCA pathway accounts for melphalan resistance in myeloma cells [18], and targeting the Fanconi anemia/BRCA pathway circumvents resistance to melphalan in myeloma cells [19].

In accordance with this point of view, it is reasonable to assume that mutations disturbing the interstrand crosslink repair function of BRCA1 and BRCA2 will make ovarian cancer or ovarian-related cancers very sensitive to melphalan. Clinical evidence supporting this assumption comes from our current presentation and a previously published article reporting an astonishing cure of a platinum-resistant *BRCA2*-related ovarian cancer by similar orally administered melphalan regimen [20].

Recently, poly(adenosine diphosphate-ribose) polymerase (PARP) inhibitor which blocks base excision repair was found to be particularly efficient in ovarian cancer deficient of homologous recombination due to *BRCA* mutation [21], and wide screening of *BRCA* mutations in both familial and sporadic ovarian cancer to expand use of PARP inhibitor has been enthusiastically advocated for improving patients’ outcomes [22]. Being worried by the heavy economic burden of this novel class of anticancer agents with special synthetic lethality capability, we are happy to learn that pharmaceutical screening has discovered the alkylators chlorambucil, melphalan, and nimustine displaying sufficient efficacy against *BRCA2*-deficient tumor cells, with melphalan and nimustine doing better than cisplatin and the PARP inhibitor olaparib *in vivo* [23].

In conclusion, based on the interesting experience deriving from this patient, similar findings in a previously published case report, and laboratory research results, we anticipate that ovarian cancer and ovarian-related cancers with *BRCA1*/*2* mutations, and even all “BRCAness” malignancy, not only correlate with high efficacy of PARP inhibitors but probably also lead to a high-potential cure by orally administered melphalan. We would like to propose that clinical trials testing the therapeutic potential of melphalan in “BRCAness” tumors be carefully designed and sophisticatedly performed. It is our hope that this oldest drug for ovarian cancer will prove itself still a faithful and powerful player in the field of cancer therapy.

## Abbreviations

CA 125: Cancer antigen 125
CA 15–3: Cancer antigen 15–3
cDNA: Complementary DNA
CK20: Cytokeratin 20
CK7: Cytokeratin 7
CLIA: Clinical Laboratory Improvement Amendments
CT: Computed tomography
ER: Estrogen receptor
FFPE: Formalin-fixed paraffin-embedded
GATA3: GATA binding protein 3
GCDFP-15: Gross cystic disease fluid protein 15
HER-2: Human epidermal growth factor receptor 2
NGS: Next-generation sequencing
PARP: Poly(adenosine diphosphate-ribose) polymerase
PAX8: Paired-box gene 8
PR: Progesterone receptor
TTF-1: Thyroid transcription factor 1
WT1: Wilms tumor 1

## Authors’ information

Fan FS is a senior attending physician in the fields of hematology, medical oncology, and hematopoietic stem cell transplantation. Yang CF is a well-trained pathologist with general interest.

## References

[1] Pavlidis N, Pentheroudakis G. Cancer of unknown primary site. Lancet. 2012;379:1428–35. 10.1016/S0140-6736(11)61178-1 22414598

[2] Fizazi K, Greco FA, Pavlidis N, Daugaard G, Oien K, Pentheroudakis G. ESMO Guidelines Committee. Cancers of unknown primary site: ESMO Clinical Practice Guidelines for diagnosis, treatment and follow-up. Ann Oncol. 2015;26(Suppl 5):v133–8. 10.1093/annonc/mdv305 26314775

[3] Kim A, Ueda Y, Naka T, Enomoto T. Therapeutic strategies in epithelial ovarian cancer. J Exp Clin Cancer Res. 2012;31:14. 10.1186/1756-9966-31-14 PMC3309949 22330607

[4] Kee Y, D'Andrea AD. Expanded roles of the Fanconi anemia pathway in preserving genomic stability. Genes Dev. 2010;24:1680–94. 10.1101/gad.1955310 PMC2922498 20713514

[5] Ettinger DS, Varadhachary GR, Handorf CR, Bowles DW, Cates JM, Chandra S, et al: NCCN Guidelines for Occult Primary (Cancer of Unknown Primary) V.2.2016. https://www.tri-kobe.org/nccn/guideline/occult/english/occult.pdf. Accessed 26 Oct 2017.

[6] Stella GM, Senetta R, Cassenti A, Ronco M, Cassoni P. Cancers of unknown primary origin: current perspectives and future therapeutic strategies. J Transl Med. 2012;10:12. 10.1186/1479-5876-10-12 PMC3315427 22272606

[7] Sangoi AR, Shrestha B, Yang G, Mego O, Beck AH. The Novel Marker GATA3 is Significantly More Sensitive than Traditional Markers Mammaglobin and GCDFP15 for Identifying Breast Cancer in Surgical and Cytology Specimens of Metastatic and Matched Primary Tumors. Appl Immunohistochem Mol Morphol. 2016;24:229–37. 10.1097/PAI.0000000000000186 PMC5610542 25906123

[8] Liliac L, Carcangiu ML, Canevari S, Căruntu ID, Ciobanu Apostol DG, Danciu M, et al. The value of PAX8 and WT1 molecules in ovarian cancer diagnosis. Romanian J Morphol Embryol. 2013;54:17–27. 23529305

[9] Acs G, Pasha T, Zhang PJ. WT1 is differentially expressed in serous, endometrioid, clear cell, and mucinous carcinomas of the peritoneum, fallopian tube, ovary, and endometrium. Int J Gynecol Pathol. 2004;23:110–8. 10.1097/00004347-200404000-00004 15084838

[10] Bian J, Li B, Kou XJ, Liu TZ, Ming L. Clinical significance of combined detection of serum tumor markers in diagnosis of patients with ovarian cancer. Asian Pac J Cancer Prev. 2013;14:6241–3. 10.7314/apjcp.2013.14.11.6241 24377511

[11] Psallidas I, Kalomenidis I, Porcel JM, Robinson BW, Stathopoulos GT. Malignant pleural effusion: from bench to bedside. Eur Respir Rev. 2016;25:189–98. 10.1183/16000617.0019-2016 PMC9487241 27246596

[12] Makar AP. Hormone therapy in epithelial ovarian cancer. Endocr Relat Cancer. 2000;7:85–93. 10.1677/erc.0.0070085 10903526

[13] Stasenko M, Plegue M, Sciallis AP, McLean K. Clinical response to antiestrogen therapy in platinum-resistant ovarian cancer patients and the role of tumor estrogen receptor expression status. Int J Gynecol Cancer. 2015;25:222–8. 10.1097/IGC.0000000000000334 25500503

[14] Cristea M, Han E, Salmon L, Morgan RJ. Practical considerations in ovarian cancer chemotherapy. Ther Adv Med Oncol. 2010;2:175–87. 10.1177/1758834010361333 PMC3126016 21789133

[15] Davis-Perry S, Hernandez E, Houck KL, Shank R. Melphalan for the treatment of patients with recurrent epithelial ovarian cancer. Am J Clin Oncol. 2003;26:429–33. 10.1097/01.COC.0000027269.06091.E9 12902900

[16] Smith JP, Rutledge F, Wharton JT. Chemotherapy of ovarian cancer. New approaches to treatment. Cancer. 1972;30:1565–71. 10.1002/1097-0142(197212)30:6<1565::aid-cncr2820300623>3.0.co;2-h 4345098

[17] Deans AJ, West SC. DNA interstrand crosslink repair and cancer. Nat Rev Cancer. 2011;11:467–80. 10.1038/nrc3088 PMC3560328 21701511

[18] Chen Q, Van der Sluis PC, Boulware D, Hazlehurst LA, Dalton WS. The FA/BRCA pathway is involved in melphalan-induced DNA interstrand cross-link repair and accounts for melphalan resistance in multiple myeloma cells. Blood. 2005;106:698–705. 10.1182/blood-2004-11-4286 PMC1895179 15802532

[19] Chen DT, Beg AA, Dalton WS. Targeting the Fanconi anemia/BRCA pathway circumvents drug resistance in multiple myeloma. Cancer Res. 2009;69:9367–75. 10.1158/0008-5472.CAN-09-2616 PMC4519834 19934314

[20] Osher DJ, Kushner YB, Arseneau J, Foulkes WD. Melphalan as a treatment for BRCA-related ovarian carcinoma: can you teach an old drug new tricks? J Clin Pathol. 2011;64:924–6. 10.1136/jcp.2010.086405 21378390

[21] Gadducci A, Guerrieri ME. PARP Inhibitors in Epithelial Ovarian Cancer: State of Art and Perspectives of Clinical Research. Anticancer Res. 2016;36:2055–64. 27127105

[22] George A, Kaye S, Banerjee S. Delivering widespread *BRCA* testing and PARP inhibition to patients with ovarian cancer. Nat Rev Clin Oncol. 2017;14:284–96. 10.1038/nrclinonc.2016.191 27958297

[23] Evers B, Schut E, van der Burg E, Braumuller TM, Egan DA, Holstege H, et al. A high-throughput pharmaceutical screen identifies compounds with specific toxicity against *BRCA2*-deficient tumors. Clin Cancer Res. 2010;16:99–108. 10.1158/1078-0432.CCR-09-2434 PMC2802735 20008842

